# Ethical religion in primary care

**DOI:** 10.1080/17571472.2017.1317407

**Published:** 2017-04-18

**Authors:** Malcolm Torry

**Affiliations:** Social Policy Department, London School of Economics, London, UK

**Keywords:** Ethics, religion, patient/practitioner relationship, principles, religious symbolism

## Abstract

Religion is increasingly significant in UK society, and is highly significant for many patients and primary care practitioners. An important task for the practitioner is to ensure that the place of religion in the patient/practitioner relationship is treated with the same ethical seriousness as every other aspect of that relationship. The article finds the ‘four principles of biomedical ethics’ to be applicable, and recent GMC guidelines to be consistent with the four principles. The article applies the four principles to the particular case of practitioners wearing religious symbolism.

## Why this matters to me?

I am a Christian minister with a research interest in how religion and religious organisations relate to society and its institutions. I am married to a GP.

## Key messages

• Religion is significant in UK society.
• Religion is significant for patients and for primary care practitioners.
• The ‘four principles of biomedical ethics’ can apply to the role of religion in the patient/practitioner relationship in the same way as they apply to other aspects of it.

## Introduction

(1) It is Ramadan, and a diabetic Muslim patient asks the practice nurse whether she should be fasting. What should he say?
(2) A female GP wears a hijab, or head scarf. During Ramadan a patient she knows to be a Muslim comes into her surgery carrying a bag of crisps. Should she say anything?
(3) A patient sees that a receptionist is wearing a lapel cross. He asks him to pray for him. Should he say that he will?

An increasingly important ethical issue facing primary care practitioners is religion: both their own, and that of their patients. This article is not primarily about the ethical aspects of the many particular religious traditions to which patients and practitioners might relate – although these will inevitably be a factor in our discussion. Rather, it is a discussion of the ethical questions that might arise in relation to the religious aspects of any relationship between a patient and a primary care practitioner.

## The question

What roles should a patient’s religion and the primary care practitioner’s religion play in a consultation?

An article in the *Journal of the American Medical Association* raises this question in an acute form. It is an account of a medical student spending time with his father, a ‘small-town Michigan physician’. When as a younger man the student had reorganised the practice’s medical notes, he was surprised by his father’s insistence that a patient’s religion was a significant part of their social history. Now they were on a home visit, during which his father suggested that they should all pray together: ‘We were there … to do exactly what Betty and John wanted at that moment: to care for their spiritual needs’ [1].

## The context: layers of diversity

But we live in increasingly secular London, which is rather different from Michigan: or is it? The institutional context will be different because primary care practitioners in the UK work in an institution in which an equality agenda and patient autonomy make religion feel like a difficult issue to handle: but that is no reason to avoid what is an increasingly important element not only in many patients’ lives but also in the lives of many practitioners.

The situation is even more complex because in fact everybody has some kind of relationship with religion; because religion will often be a (generally unspoken) factor in the relationship between a patient and a primary care practitioner; and because religion is increasingly significant in every society, including secularising ones. The religious aspects of a patient/practitioner relationship will be influenced by the religious currents flowing through our diverse society, and in turn that relationship cannot help affecting our society’s relationship with religion.

When we ask how we are to understand religion’s place in a consultation between a patient and a primary care practitioner, the first thing that we have to say is that no general answer can be given. Every country, every city, and every community, contains a different mix of religious affiliations.^1^ Within each faith community there will be major divisions, such as those between Shia and Sunni Muslims, Orthodox and Reform Jews, and Roman Catholic, Eastern Orthodox, and Protestant Christians. Further divisions are caused by cultural differences, so that it might be better to think in terms of ethno-religious groups in society than in terms of purely religious groups [2]. Yet another layer of diversity is of levels of religious adherence [3]^2^: participation in religion is a spectrum, with at one end census self-identification and at the other active participation in a congregation. And within any congregation there will be major differences between different members’ beliefs and practices [4,5]. There is no such thing as religion. There are religions, and there are the diverse religious beliefs and practices of individuals.

The complex context that I have outlined means that nowhere are there settled relationships between governments, civil society, and faith communities; that in increasing numbers of places the religious context in which primary care practitioners are working might be changing quite fast and can be difficult to negotiate; that every patient is different in relation to their religious understanding and practice; that every practitioner is different in relation to their religion; that every patient/practitioner relationship takes place in a situation of deep and changing religious diversity; and that very little is predictable. An essential piece of advice must therefore be this: Never assume; listen carefully; ask if necessary.

## The patient-practitioner relationship

I shall construct the rest of this discussion around the ‘four principles of biomedical ethics’: respect for autonomy, beneficence, non-maleficence, and justice [6,7].^3^

## Respect for autonomy

Every patient will have their own unique relationship with one or more religions, and their own preferences in relation to the privacy or otherwise of that relationship. This means that in normal circumstances the practitioner has no right to enquire about a patient’s religious beliefs or practices. It would of course be both ethical and essential to participate in a discussion of religious belief and/or practice if a patient were to initiate such a discussion. During such discussions the practitioner should make no assumptions, but should simply receive information from the patient. If it appears to the practitioner that the religious information being offered might be relevant to the patient’s healthcare, then questions for elucidation would not compromise the patient’s autonomy: but it would not be the practitioner’s role to seek irrelevant information, or to probe beyond the patient’s clear willingness to volunteer information.

The situations in which it would be ethical for a practitioner to volunteer information about their own religious commitment, beliefs and practice (including beliefs about the falsity or irrelevance of religion) will be extremely rare. If a patient were to ask a practitioner whether they shared and practised their religion, and were then to ask the practitioner to join them in a religious practice such as prayer, then the patient’s autonomy would require the practitioner to do as requested if the practitioner believed that to do so might be beneficial to the patient and would not cause any harm.

Respect for the autonomy of the patient might require the practitioner to assist the patient to make their own informed choices. If it is clear from information volunteered by the patient that a religious practice might damage their health – for instance, if fasting might delay recovery or might jeopardise health – then the practitioner will need to provide the patient with sufficient information to make their own decision as to whether or not to continue to undertake the religious practice. If the patient recognises the authority of a religious organisation or functionary, then encouraging them to consult that functionary or organisation will count as an encouragement to them to exercise their autonomy.

## Beneficence

The only criterion here is the benefit of the patient, and as only the patient will know the relationship between their religious beliefs and practices and the other areas of their lives, only the patient will be able to evaluate the health benefits and disbenefits of their relationship with their religion. So as we have recognised, the patient might invite a co-religionist practitioner to join them in a religious practice. The invitation will indicate that the patient believes that such an activity will be beneficial. The practitioner can help to ensure that the activity will be beneficial by not making any assumptions about the particular religious beliefs of the patient, but instead allowing the patient to determine the nature and content of the religious activity. If the practitioner’s own religious beliefs will permit them to undertake the activity, and if they judge that undertaking it will be beneficial to the patient, then the ethical decision is to agree to join the patient in the religious activity.

As we have already recognised, the requirements of beneficence and of recognising the patient’s autonomy here overlap. They also overlap if the patient asks advice about the relationship between their health (broadly defined) and their religious beliefs and practices. If the request comes from the patient, and if the practitioner makes no assumptions about the patient’s beliefs and practices, and only asks questions in the cause of seeking elucidation of the patient’s statements, then the patient’s autonomy can be secured; and their autonomy will continue to be secured if any advice requested relates only to the patient’s health and to the religious information offered by the patient.

It is an interesting question as to how to interpret the requirement that ‘the healthcare professional should act in a way that benefits the patient’. Clearly beneficence is achieved if the patient’s physical and/or mental health benefits from a patient-initiated discussion of their religious faith and practice: but does the ‘beneficence’ requirement require the practitioner to seek to benefit the patient’s relationship with their religion? The answer has to be ‘yes’. It is the patient’s benefit that is sought by the ethical principle, and not simply some aspect of the patient. Here great care will be needed, because it will never be clear to the practitioner precisely how anything that they might say might benefit the religious aspects of the patient’s life. The practitioner will remain on ethically safe ground if they are discussing religious matters at the invitation of the patient, and if they avoid detailed leading questions and only offer open questions and options for the patient to consider. ‘Is there anyone it might be useful to consult about that?’ rather than ‘Do you think you should consult your priest about that?’ and ‘Which would be better for you: to fast or not to fast?’ rather than ‘Might your health benefit from not fasting?’ or ‘What would your imam think about you not fasting?’

No part of a patient’s life can be isolated from any other part, so although the healthcare practitioner’s chief concern is with the patient’s physical and mental health, in order to benefit those, and in order to benefit the patient as a whole, the practitioner might have to involve themselves with the patient’s religion. Provided that the only considerations in the practitioner’s mind are the patient’s autonomy and the patient’s benefit, this should never be difficult. It does not require any religious knowledge on the part of the practitioner, because anything relevant will come from the patient. All it requires is sufficient empathy to understand how the patient relates to relevant aspects of their own religious belief and practice.

## Non-maleficence

This is the obverse of beneficence, and in some discussions of the four principles it is coupled with it [7], because in satisfying beneficence the practitioner might believe that they are satisfying non-maleficence. However, in the case of the practitioner’s relationship with the patient’s religion, it might be helpful to keep the two apart and to ask how the practitioner can avoid doing harm to the patient.

Here again the relationship with the requirement for the patient’s autonomy is crucial. If the patient leads any discussion of their religion, if any practitioner questions are for elucidation only, and if all that the practitioner offers is open questions and options for the patient to consider, then the patient’s autonomy will be maintained and the practitioner will not harm the patient. Similarly, if any request for information about the practitioner’s religious belief and practice, and any request for participation in a religious practice, are entirely led by the patient, then harm should be avoided and the patient’s autonomy secured.

But here a question of timescale emerges. It is always possible for a treatment to benefit the patient’s health in the short term and to damage it in the longer term. The same is true of any involvement of the practitioner in the patient’s relationship with their religion. To take an example: A patient might ask a practitioner what their religion is. If it is different from their own, then they might still ask the practitioner to participate in a religious activity such as prayer, and the practitioner might be comfortable with doing that. However, to cross religious boundaries is rarely universally acceptable in a faith community, and on the assumption that the patient might speak about a prayer offered for them by someone of a different faith, the practitioner ought to ask themselves whether disbenefit might accrue both for the patient and for other patients of the practice. Whenever there is the slightest doubt in a practitioner’s mind about carrying out a requested religious activity, particularly one that belongs to a faith community other than their own, then the practitioner should go with their doubt, should gently decline, and should suggest that the patient should seek out a trusted co-religionist to pray with them.

Religious issues can be some of the most complex ethical issues that a practitioner finds themselves involved with. The easiest approach is to steer clear of them, and this might look like the best way to avoid maleficence: but this strategy also denies to the patient the potential benefit that might accrue from a practitioner’s careful engagement with their religion. Judicious engagement can ensure both benefit and non-maleficence.

## Justice

Provided that a practitioner does not employ large amounts of consulting time relating beneficially to a patient’s relationship between their health and their religion, the practitioner will not deny such attention to another patient, so there is unlikely to be an issue of allocative justice to consider. This leaves the question as to whether the practitioner is treating similar patients in similar ways. We have already discussed an essential difference between the way in which a practitioner might participate in a religious practice with a patient whose religion they share and the way in which they might not share in a religious practice with a patient whose religion they do not share; but apart from that, the ways in which a practitioner might relate to a patient’s religion that we have discussed will apply equally to a patient whose religion the practitioner shares and to a patient whose religion they do not. As long as the practitioner maintains this equality of approach, the principle of justice will be served.

## General Medical Council guidelines

Following a complaint that a practitioner had imposed his religious views on a patient, and the GMC issuing a formal warning to the General Practitioner concerned,^4^ the UK’s General Medical Council extracted paragraphs from its *Good Medical Practice* guide under the title ‘Personal Beliefs and Medical Practice (2013)’ [8] and also issued a detailed discussion of the guidelines:

You must … adequately assess the patient’s conditions, taking account of their history (including the symptoms and psychological, spiritual, social and cultural factors), their views and values; where necessary, examine the patient; … You must treat patients fairly and with respect whatever their life choices and beliefs. … You must explain to patients if you have a conscientious objection to a particular procedure. You must tell them about their right to see another doctor … You must not express your personal beliefs (including political, religious and moral beliefs) to patients in ways that exploit their vulnerability or are likely to cause them distress. … The investigations or treatment you provide or arrange must be based on the assessment you and your patient make of their needs and priorities, … You must not refuse or delay treatment because you believe that a patient’s actions or lifestyle have contributed to their condition. … You must not unfairly discriminate against patients or colleagues by allowing your personal views to affect your professional relationships or the treatment you provide or arrange.^5^

As the reader will see, the practitioner will satisfy these provisions if they follow the four principles of biomedical ethics in the ways that I have suggested.

## Religious symbolism

A particularly fraught recent issue has been that of healthcare workers wearing symbolism that might declare their religion. Is this issue amenable to our ‘four principles’ framework for ethical religion?

In 2012, the European Court of Human Rights heard two similar complaints. Both Nadia Eweida, a British Airways staff member, and Shirley Chaplin, a geriatric nurse, had complained that their employers had breached their human right to free expression of their religion by asking them to remove chains to which crosses were attached. The court found that British Airways had been wrong to ask Ms. Eweida to remove the cross. The airline changed its policy.^6^ However, it found that Ms. Chaplin’s health authority had been right to ask her to remove hers because they regarded the chain on which the cross was hung to be a safety risk. She had declined to wear a lapel cross instead [9]. In January 2015 the decision was upheld on appeal.^7^

The important factor here is that the health authority recognised its staff member’s right to express her religion by wearing a lapel cross. This is surely right. To wear a discrete lapel cross – or any other discrete and safe religious symbolism – would cohere with the framework that we have developed. It would not compromise the patient’s autonomy; it would not exploit a patient’s vulnerability or cause them distress, so it would not be maleficent; it would fulfil the practitioner’s human right to express their religion, and would therefore be an example of justice (and it would not be unjust to the patient); and it might also be beneficent, in the sense that patients of the same or a different religion might find it helpful to know that the practitioner practices a religion and what that religion is so that they might have the option of relating to the practitioner’s faith if that is what they choose to do.

## Conclusion

In one sense of course religious issues are not like any others: but because for the patient their religion might be intimately connected with every other aspect of their being, it might be important for an explicitly ‘spiritual assessment’ to be undertaken as a contribution towards an understanding of the patient as a whole person. As a Canadian General Practitioner suggests, such a spiritual assessment

need not be an intrusive or invasive process. … It might … give us a richer understanding of how individual patients interpret the challenges they face. It might assist the therapeutic alliance in useful and unexpected ways. The very act of acknowledging a spiritual dimension in health allows the patient to know that we are sensitive to needs, aspiration, and concerns in this arena. [10]

We might be encouraged to undertake a more explicit engagement with religion in the context of the patient/practitioner relationship by the fact that it is perfectly possible to apply the four principles of biomedical ethics to religious aspects of the patient/practitioner relationship, and that no additional guidelines need to be applied for the practitioner to ensure that they are acting ethically. In this sense religious issues are no different from any other aspect of a patient’s life, and no practitioner should find it difficult to behave ethically in relation to their patients’ religious beliefs and practices.

If a practitioner follows the rule ‘Never assume; listen carefully; ask if necessary’ and keeps in mind the four principles, then any consultation should be able to go in a direction beneficial to the patient.

So in answer to the three questions at the beginning of this article:

(1) The nurse should hold a discussion with the patient, and ask whether she believed her religion to require fasting of someone with her medical condition. The patient might volunteer the information that fasting would not be necessary under those circumstances, or might say that she wasn’t sure: in which case the nurse could legitimately say that he thought that it might not be necessary. He might then ask if there was someone the patient could consult in order to obtain accurate information. The patient might suggest that she should consult the Imam of the mosque that she attended.
(2) The GP should say nothing.
(3) The receptionist could legitimately promise to pray for the patient if he wished to do that and there was no obvious reason not to.

## Disclosure statement

No potential conflict of interest was reported by the author.

## Ethical approval

Ethical approval was not sought because this was not a research project.

## Notes

1. It would be impossible in this short article to do justice to the religious context of every country from which its readers might come, so just to take two examples: In England and Wales the 2011 census figures show that 59.3% of the population declare themselves to be Christians, 4.8% Muslims, other religions add up to 3.6%, 7.2% do not state their religion (the census question was voluntary), and 25.1% state ‘no religion’. India’s 2011 census showed that 78.35% of the population practice Hinduism, 14.88% Islam, and 6.77% a wide variety of other religions, including Christianity, Sikhism, Buddhism, Jainism, Zoroastrianism, Judaism, and the Baha’i faith. Sources: census 2011, Office for National Statistics; and http://indianexpress.com/article/india/india-others/census-hindu-share-dips-below-80-muslim-share-grows-but-slower/. This press report is the only record so far of the Indian 2011 census results on religious affiliation. Official figures have not been published.

2. A UK time use survey suggests that less than 7% of the population of England and Wales attend a church service on any given Sunday. This is well below the 59.3% who in 2011 declared themselves to be Christians.

3. http://www.ukcen.net/index.php/ethical_issues/ethical_frameworks/the_four_principles_of_biomedical_ethics.

4. http://www.gmc-uk.org/news/13333.asp.

5. http://www.gmc-uk.org/static/documents/content/Personal_beliefs_and_medical_practice.pdf.

6. www.telegraph.co.uk/news/religion/9802067/Christian-wins-right-to-wear-cross-at-work.html.

7. http://hudoc.echr.coe.int/webservices/content/pdf/003-4221189-5014359.
